# [ ^18^F]PSMA‐1007 PET–Based Recurrence Site Distribution and Predictors of Metastatic Disease in Patients With Biochemical Recurrence of Prostate Cancer

**DOI:** 10.1111/iju.70587

**Published:** 2026-07-31

**Authors:** Koji Hatano, Tadashi Watabe, Takero Hirata, Masatoshi Konishi, Tomohiro Kanaki, Masaru Tani, Toshiki Oka, Yu Ishizuya, Takuji Hayashi, Yoshiyuki Yamamoto, Taigo Kato, Atsunari Kawashima, Kazutoshi Fujita, Motohide Uemura, Frederik L. Giesel, Norio Nonomura

**Affiliations:** ^1^ Department of Urology The University of Osaka Graduate School of Medicine Suita Japan; ^2^ Department of Radiology The University of Osaka Graduate School of Medicine Suita Japan; ^3^ Institute for Radiation Sciences The University of Osaka Suita Japan; ^4^ Department of Radiation Oncology The University of Osaka Graduate School of Medicine Suita Japan; ^5^ Department of Urology Kindai University Faculty of Medicine Sakai Japan; ^6^ Department of Urology Fukushima Medical University School of Medicine Fukushima Japan; ^7^ Department of Urology Iwase General Hospital Sukagawa Japan; ^8^ Department of Nuclear Medicine Medical Faculty and University Hospital Düsseldorf, Heinrich‐Heine‐University Düsseldorf Germany

**Keywords:** metastasis, positron‐emission tomography, prostate cancer, prostate‐specific antigen, prostate‐specific membrane antigen

## Abstract

**Objectives:**

Prostate‐specific membrane antigen positron emission tomography (PSMA‐PET) is a sensitive imaging modality for detecting recurrent prostate cancer following definitive local therapy. However, evidence on ^18^F‐labeled PSMA tracers in Japanese patients with biochemical recurrence (BCR) remains limited. The aim of this study was to examine the recurrence site distribution by [^18^F]PSMA‐1007 PET in patients with BCR and identify factors associated with metastatic disease.

**Methods:**

We retrospectively analyzed 129 patients with prostate cancer who developed BCR after definitive local therapy and underwent [^18^F]PSMA‐1007 PET. The primary endpoint was the distribution of recurrence sites detected by PSMA‐PET. Secondary endpoints included clinicopathological factors associated with metastatic disease. Exploratory endpoints involved outcomes of lesion‐directed therapy following PSMA‐PET.

**Results:**

PSMA‐PET identified recurrent lesions in 83% (*n* = 107), including local recurrence in 40% (*n* = 51), lymph node metastasis in 43% (*n* = 55), and bone metastasis in 26% (*n* = 33); sites were not mutually exclusive. Metastatic lesions were detectable even at low prostate‐specific antigen (PSA) levels (≤ 1.0 ng/mL). In multivariate analysis, Grade Group ≥ 4 was independently associated with metastatic disease (odds ratio [OR], 2.65; 95% confidence interval [CI], 1.06–6.77), whereas a PSA doubling time ≥ 8.7 months was independently associated with a lower likelihood of metastasis (OR, 0.12; 95% CI, 0.05–0.27). Among 55 patients who underwent lesion‐directed therapy, PSA declines ≥ 90% were observed in 60% of cases.

**Conclusions:**

In this Japanese cohort with BCR, [^18^F]PSMA‐1007 PET delineated recurrence patterns and identified clinicopathological factors associated with metastatic disease. PSMA‐PET may provide clinically relevant information to support individualized salvage treatment strategies.

## 1 Introduction

Prostate cancer is among the most common malignancies affecting men worldwide, and definitive local therapies such as radical prostatectomy and radiation therapy achieve favorable oncologic outcomes in many patients. Nevertheless, a considerable proportion of patients experience biochemical recurrence (BCR), defined by an increase in prostate‐specific antigen (PSA) levels during follow‐up [1, 2, 3]. The management of BCR remains clinically challenging because accurate localization of recurrent disease is essential for selecting appropriate salvage treatment strategies. Conventional imaging modalities, including computed tomography (CT) and bone scintigraphy, have limited sensitivity for detecting small‐volume disease—particularly at low PSA levels [4]—which may lead to suboptimal treatment selection.

Prostate‐specific membrane antigen positron emission tomography (PSMA‐PET) has emerged as a highly sensitive imaging modality for detecting recurrent and metastatic prostate cancer [5, 6, 7, 8, 9, 10]. Among available tracers, [^18^F]PSMA‐1007 offers distinct advantages, including high spatial resolution and minimal urinary excretion, facilitating the detection of local recurrence and pelvic lesions [8, 9, 10]. A previous study by Watabe et al. at The University of Osaka demonstrated the diagnostic performance of [^18^F]PSMA‐1007 PET in patients with BCR [10], and subsequent studies have described the patterns of recurrent disease in this setting [11, 12, 13]. However, clinical factors associated with metastatic findings on [^18^F]PSMA‐1007 PET have not been fully elucidated. In Japan, where PSMA‐PET is not routinely reimbursed for BCR, identifying patients at an increased risk of metastatic disease may help optimize imaging and salvage treatment strategies.

Therefore, this study aimed to assess the distribution of recurrence sites detected by [^18^F]PSMA‐1007 PET in patients with BCR after definitive local therapy who had no evidence of metastasis on conventional imaging. We also evaluated clinicopathological factors associated with metastatic recurrence and outcomes of lesion‐directed therapy following PSMA‐PET.

## 2 Methods

### 2.1 Study Design and Patient Selection

**FIGURE 1:**
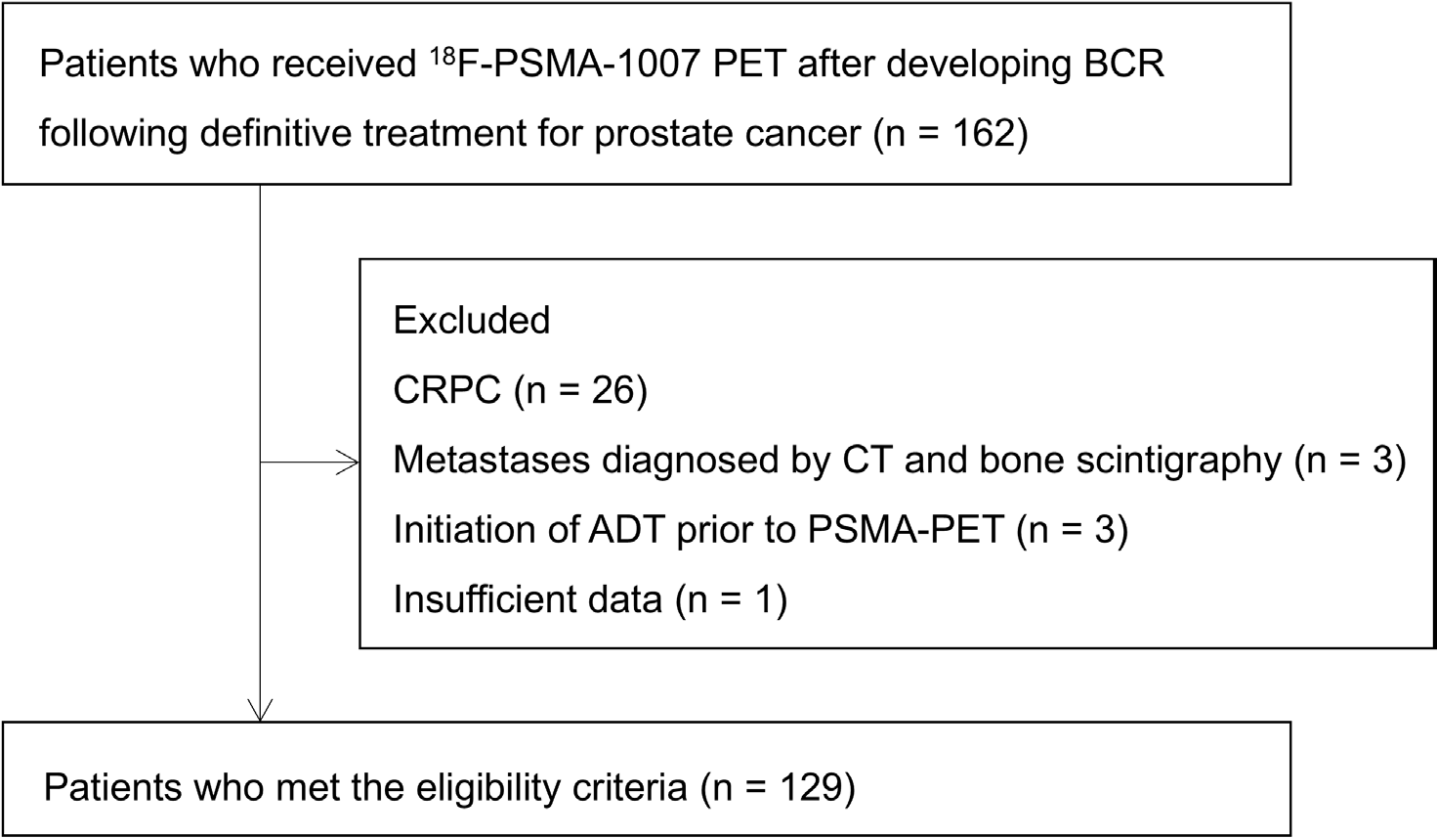
Flowchart of patient selection. ADT, androgen deprivation therapy; BCR, biochemical recurrence; CRPC, castration‐resistant prostate cancer; CT, computed tomography.

This retrospective study included patients with prostate cancer who developed BCR after definitive local therapy and underwent [^18^F]PSMA‐1007 PET between October 2019 and February 2024 as part of a clinical research protocol [10]. BCR was defined as a PSA level of ≥ 0.2 ng/mL at least 6 weeks after radical prostatectomy or a rise of ≥ 2.0 ng/mL above the nadir after radiotherapy, according to the ASTRO–Phoenix consensus definition [14]. Patients with persistently elevated or rising PSA levels after definitive local therapy were eligible. Exclusion criteria included castration‐resistant prostate cancer (CRPC), metastatic disease identified on conventional imaging, prior androgen deprivation therapy (ADT), or insufficient clinical data. Of 162 patients screened, 33 were excluded (26 with CRPC, 3 with metastases identified on conventional imaging, 3 with prior ADT, and 1 with insufficient data), leaving 129 patients for the final analysis (Figure 1).

### 2.2 [ ^18^F]PSMA‐1007 PET/CT Imaging

[^18^F]PSMA‐1007 PET/CT imaging was performed in accordance with a previously described protocol [10]. Briefly, [^18^F]PSMA‐1007 was synthesized using an automated radiosynthesis system (MPS200, Sumitomo Heavy Industries, Japan). Patients received an intravenous injection of [^18^F]PSMA‐1007 (249 ± 33 MBq), and PET/CT imaging was initiated approximately 60 min after tracer administration. Scans were acquired using a Discovery 710 scanner (GE Healthcare, Milwaukee, WI, USA). PET images were independently interpreted by two board‐certified nuclear medicine physicians blinded to clinical outcomes. Discrepancies were resolved by a senior nuclear medicine specialist experienced in PSMA‐PET interpretation. The detected lesions were categorized as local recurrence (prostate or prostate bed), lymph node metastasis, or bone metastasis. The detection rate was defined as the proportion of patients with at least one PSMA‐PET‐positive lesion, and patients were classified according to the most advanced site of disease.

### 2.3 Study Endpoints

The primary endpoint was the distribution of recurrence sites detected by PSMA‐PET. The detection rate was further analyzed according to PSA levels at BCR, stratified as < 0.5, 0.5–1.0, and > 1.0 ng/mL. The secondary endpoint was the identification of clinicopathological factors associated with metastatic disease, including bone and lymph node metastases, on PSMA‐PET imaging. Evaluated factors included age, pathological grade, clinical stage at diagnosis, type of definitive local therapy, PSA level at BCR, and PSA doubling time (PSA‐DT) at BCR. Pathological findings were based on biopsy specimens in both the radical prostatectomy and radiotherapy groups. PSA‐DT was calculated using a previously described method [15]. Sensitivity analyses were conducted using alternative PSA‐DT cutoffs ranging from 6 to 12 months. The exploratory endpoints were the evaluation of treatment selection patterns following PSMA‐PET and the clinical outcomes of lesion‐directed therapy.

### 2.4 Lesion‐Directed Therapy and Outcome Assessment

Patients with PSMA‐PET–detected localized or oligometastatic recurrence (≤ 5 lesions) were considered for salvage therapy after multidisciplinary discussion. Local and bone lesions were treated with stereotactic body radiation therapy (SBRT) or conventionally fractionated volumetric modulated arc therapy, and lymph node lesions were managed with SBRT or laparoscopic lymph node dissection. Salvage prostate irradiation after prior radiotherapy was performed only for biopsy‐confirmed local recurrence. Clinical outcomes were exploratorily evaluated in patients who did not receive concomitant ADT. Of the 59 patients who underwent salvage therapy, 55 were included after excluding those who received ADT (*n* = 3) or had insufficient follow‐up (*n* = 1). Biochemical response was assessed using PSA levels, and PSA progression‐free survival (PSA‐PFS) was defined as the time from salvage therapy to biochemical progression or last follow‐up.

### 2.5 Statistical Analysis

Factors associated with metastatic recurrence were evaluated using logistic regression analysis. PSA‐PFS was estimated using the Kaplan–Meier method. All statistical tests were two‐sided, and a *p*‐value < 0.05 was considered statistically significant. Statistical analyses were conducted using JMP Student Edition, version 18 (SAS Institute Inc., Cary, NC, USA).

### 2.6 Ethical Considerations

This study was conducted in accordance with the principles of the Declaration of Helsinki and was approved by the Institutional Review Board of Osaka University Hospital (Approval Nos. 19 066 and 13 397–24). All patients provided written informed consent for participation in the clinical study.

## 3 Results

### 3.1 Patient Characteristics

Baseline characteristics of the 129 eligible patients are summarized in Table 1. The median age was 72 years (interquartile range [IQR], 67–76). Grade Group ≤ 3 was observed in 81 patients (63%), whereas Grade Group ≥ 4 was identified in 48 patients (37%). Definitive treatment consisted of radical prostatectomy in 79 patients (61%) and radiation therapy in 50 patients (39%). The median PSA level at BCR was 2.2 ng/mL (IQR, 0.6–3.6), and the median PSA‐DT at BCR was 8.7 months (IQR, 4.4–17.3).

**TABLE 1:** Patient characteristics (*n* = 129).

Age, Median (IQR)	72 (67–76)
Pathological grade, *n* (%)	
GG1	18 (14)
GG2	26 (20)
GG3	37 (29)
GG4	29 (22)
GG5	19 (15)
Clinical stage at diagnosis, *n* (%)	
T1	16 (12)
T2	78 (60)
T3	30 (23)
T4	1 (1)
Tx	4 (3)
Primary therapy, *n* (%)	
Radical prostatectomy	79 (61)
Radiation therapy	50 (39)
PSA value at BCR (ng/mL), Median (IQR)	2.2 (0.6–3.6)
PSA‐DT at BCR (months), Median (IQR)	8.7 (4.4–17.3)

Abbreviations: BCR, biochemical recurrence; GG, Grade Group; IQR, interquartile range; PSA, prostate specific antigen; PSA‐DT, PSA‐doubling time.

### 3.2 PSMA‐PET Detection Rate

**FIGURE 2:**
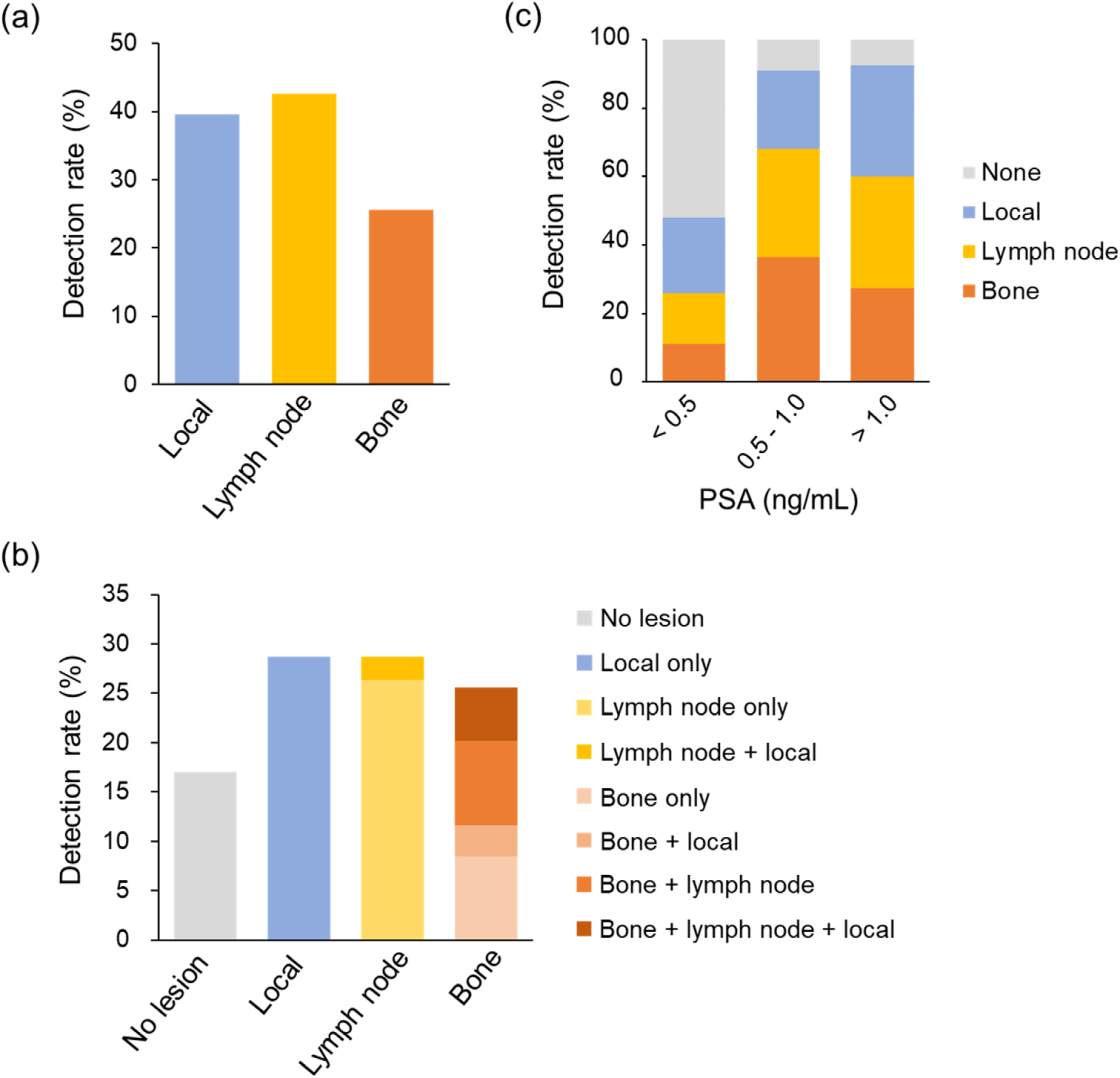
[^18^F]PSMA‐1007 PET detection rates according to recurrence patterns. (a) Detection rates, categorized as local recurrence, lymph node metastasis, and bone metastasis. (b) Detection rates based on the most advanced site of disease in patients with multiple lesions. (c) Detection rates stratified by prostate‐specific antigen (PSA) levels at biochemical recurrence: < 0.5 ng/mL, 0.5–1.0 ng/mL, and > 1.0 ng/mL.

PSMA‐PET identified recurrent disease in 107 of 129 patients (83%). The sites of recurrence included local recurrence in 51 patients (40%), lymph node metastases in 55 (43%), and bone metastases in 33 (26%), with overlapping sites in some patients (Figure 2a). Classification according to the most advanced site revealed bone metastases in 33 patients (26%), lymph node metastases in 37 (29%), local recurrence in 37 (29%), and no detectable disease in 22 (17%) (Figure 2b). When stratified by PSA level at biochemical recurrence, the overall detection rate was 48% at < 0.5 ng/mL, 91% at 0.5–1.0 ng/mL, and 93% at > 1.0 ng/mL (Figure 2c; Table S1). The metastasis detection rate, combining bone and lymph node metastases, was 26% at < 0.5 ng/mL, 68% at 0.5–1.0 ng/mL, and 60% at > 1.0 ng/mL, indicating that metastatic disease could be detected even at relatively low PSA levels.

### 3.3 Clinicopathological Factors Associated With Metastatic Disease on PSMA‐PET

**FIGURE 3:**
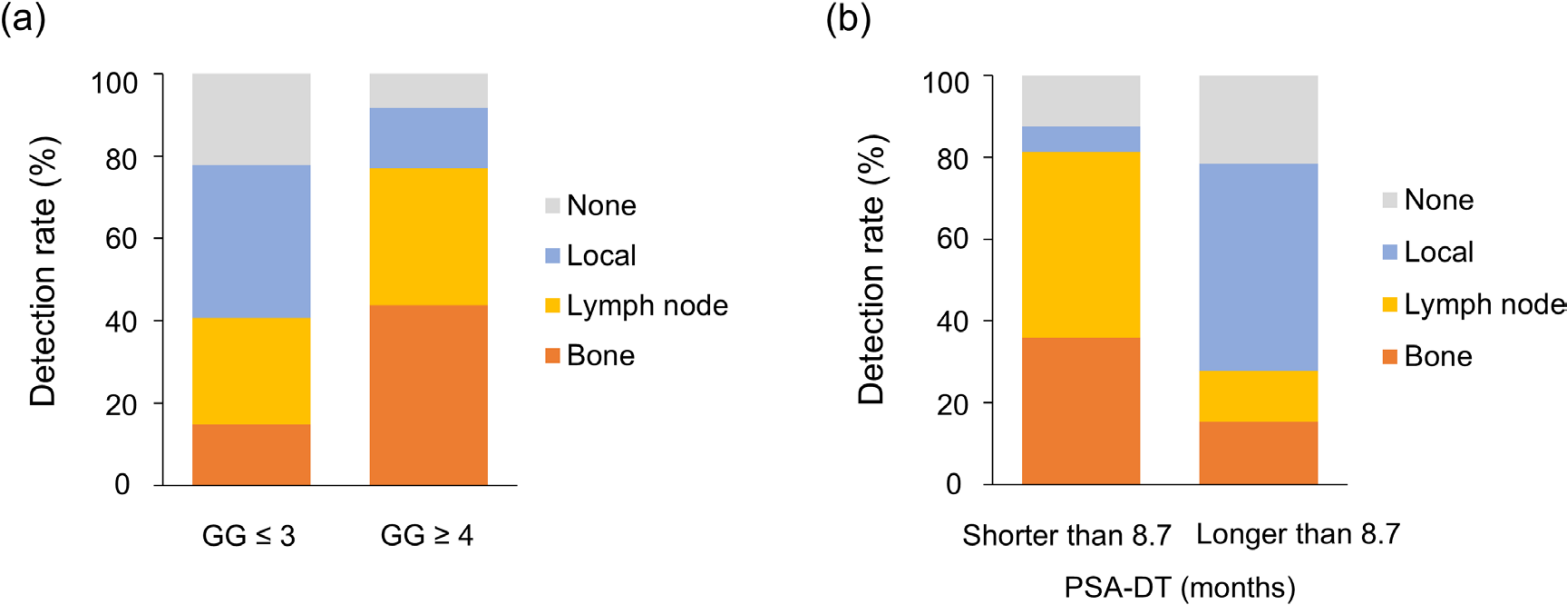
[^18^F]PSMA‐1007 PET detection rates stratified by (a) Grade Group (GG) and (b) PSA doubling time (PSA‐DT), categorized as no detectable disease, local recurrence, lymph node metastasis, and bone metastasis.

Clinicopathological factors associated with metastatic disease detected by PSMA‐PET were analyzed using logistic regression (Table 2). In univariate analysis, patients with Grade Group ≥ 4 had a significantly higher likelihood of metastatic disease compared with those with Grade Group ≤ 3 (odds ratio [OR], 4.89; 95% confidence interval [CI], 2.24–11.35). PSA‐DT at BCR was dichotomized at the median value of 8.7 months; a PSA‐DT ≥ 8.7 months was inversely associated with metastatic disease (OR, 0.09; 95% CI, 0.04–0.20). PSA‐DT at BCR was also inversely associated with metastatic disease when analyzed as a continuous variable (OR: 0.85; 95% CI: 0.79–0.90) (Table S2). In multivariate analysis (model 1), Grade Group ≥ 4 remained an independent risk factor for metastatic disease (OR, 2.65; 95% CI, 1.06–6.77), whereas a PSA‐DT ≥ 8.7 months was independently associated with a lower likelihood of metastasis (OR, 0.12; 95% CI, 0.05–0.27). Furthermore, in multivariable model 2, which included pathological Grade Group, PSA‐DT, and primary treatment, Grade Group ≥ 4 and shorter PSA‐DT remained independently associated with metastatic disease, whereas primary treatment did not (Table 2). Consistent with these findings, metastatic disease was more frequently observed in patients with Grade Group ≥ 4 than in those with Grade Group ≤ 3 (77% vs. 41%, *p* < 0.01) (Figure 3a). Similarly, patients with a PSA‐DT < 8.7 months had a significantly higher rate of metastatic disease compared with those with a PSA‐DT ≥ 8.7 months (81% vs. 28%, *p* < 0.001) (Figure 3b). The association between shorter PSA‐DT and metastatic disease remained consistent across alternative PSA‐DT cutoffs ranging from 6 to 12 months (Table S3).

**TABLE 2:** Univariate and multivariate logistic regression models for predicting metastatic disease.

	Univariate				Multivariate (model 1)				Multivariate (model 2)			
	OR	95% CI		*p*	OR	95% CI		*p*	OR	95% CI		*p*
		Lower	Upper			Lower	Upper			Lower	Upper	
Age (≥ 72 years vs. < 72 years)	0.90	0.45	1.81	0.77								
Pathological grade (≥ GG4 vs. ≤ GG3)	4.89	2.24	11.35	< 0.001	2.65	1.06	6.77	0.04	2.74	1.06	7.24	0.04
Clinical stage at diagnosis (≥ cT3 vs. ≤ cT2)	1.07	0.47	2.44	0.87								
Primary therapy (prostatectomy vs. RT)	1.51	0.74	3.10	0.26					0.88	0.36	2.13	0.78
PSA value at BCR (≥ 2.2 vs. < 2.2 ng/mL)	1.51	0.75	3.04	0.25								
PSA‐DT at BCR (≥ 8.7 vs. < 8.7 months)	0.09	0.04	0.20	< 0.001	0.12	0.05	0.27	< 0.001	0.11	0.05	0.27	< 0.001

Abbreviations: BCR, biochemical recurrence; CI, confidence interval; GG, Grade Group; OR, odds ratio; PSA, prostate specific antigen; PSA‐DT, PSA‐doubling time; RT, radiation therapy.

### 3.4 Treatment Selection Following PSMA‐PET and Outcomes of Lesion‐Directed Therapy

**FIGURE 4:**
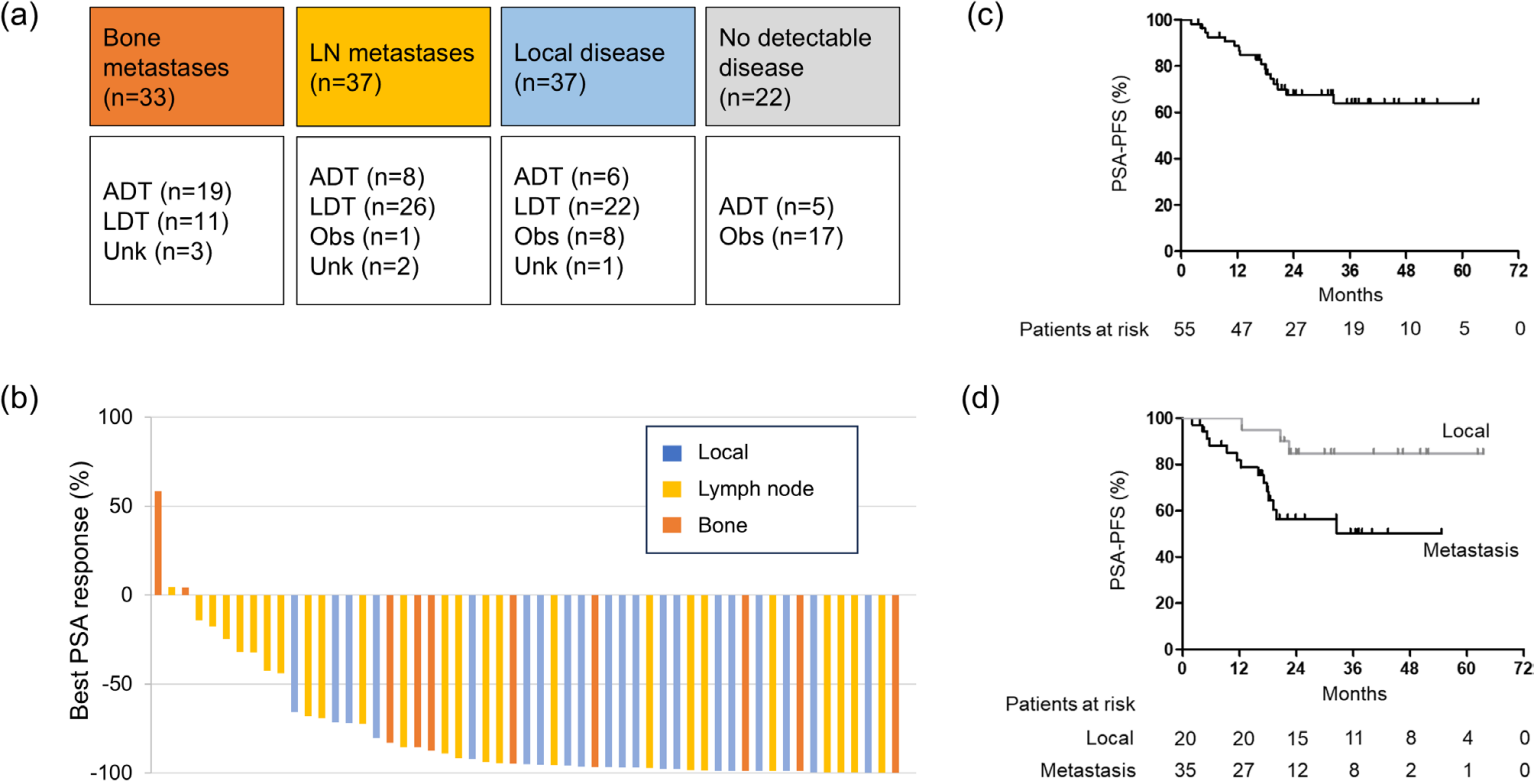
Treatment selection following [^18^F]PSMA‐1007 PET and outcomes of lesion‐directed therapy. (a) Treatment selection after imaging, categorized as lesion‐directed therapy (LDT), androgen deprivation therapy (ADT), observation (Obs), and unknown (Unk). (b) Maximum prostate‐specific antigen (PSA) decline after lesion‐directed therapies in patients with biochemical recurrence, categorized as local recurrence, lymph node metastasis, and bone metastasis. (c) Kaplan–Meier estimates of PSA progression‐free survival (PSA‐PFS) in patients treated with lesion‐directed therapy. (d) PSA‐PFS stratified by disease status on PET, shown separately for patients with localized recurrence and those with metastatic disease.

**FIGURE 5:**
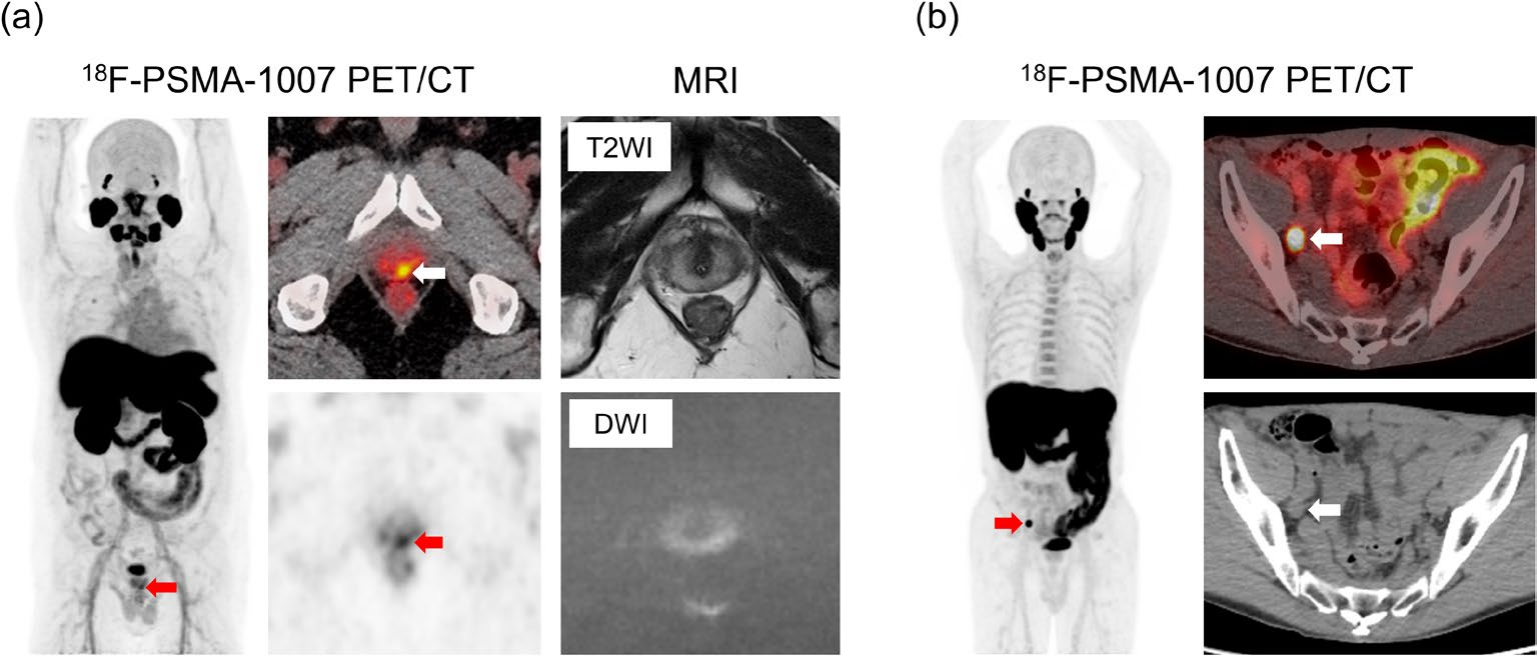
Representative cases of [^18^F]PSMA‐1007 PET–guided lesion‐directed therapy. (a) A patient with biochemical recurrence (BCR) after heavy ion radiotherapy (PSA at PET: 3.57 ng/mL). Focal uptake in the prostate was detected on PSMA‐PET (arrows) and confirmed as recurrence by biopsy, while magnetic resonance imaging (MRI) was negative. CyberKnife‐based salvage radiotherapy led to a PSA decline to 0.08 ng/mL, with durable disease control for 56 months. DWI, diffusion‐weighted imaging; T2WI, T2‐weighted imaging. (b) A patient with BCR after CyberKnife radiotherapy (PSA at PET: 5.10 ng/mL). PSMA‐PET identified a solitary pelvic lymph node metastasis (arrows). Salvage laparoscopic lymph node dissection resulted in a PSA decrease to 0.04 ng/mL. Histopathology confirmed nodal metastasis, and the patient remains recurrence‐free at 44 months.

Treatment selection following PSMA‐PET imaging is summarized in Figure 4a. Overall, lesion‐directed therapy was chosen for 59 patients (46%), ADT for 38 patients (29%), and active observation for 26 patients (20%). Exploratory outcomes of lesion‐directed therapy were evaluated in 55 patients without concomitant ADT, with a median follow‐up of 23 months (IQR, 17–38), including 35 with metastatic disease and 20 with local recurrence. A decline in PSA levels of ≥ 50% was achieved in 45 patients (82%), and a decline of ≥ 90% was observed in 33 patients (60%) (Figure 4b). The overall PSA‐PFS rate was 85% at 1 year and 68% at 2 years (Figure 4c). When stratified by disease status, PSA‐PFS for patients with localized recurrence was 95% at 1 year and 85% at 2 years, whereas that for patients with metastatic disease was 79% at 1 year and 57% at 2 years (Figure 4d). In a subset of patients, lesion‐directed therapies following PSMA‐PET resulted in sustained PSA reductions and delayed initiation of ADT. Figure 5 shows the representative cases.

## 4 Discussion

In this study, we assessed the distribution of recurrence sites detected by [^18^F]PSMA‐1007 PET in patients with BCR after definitive local therapy for prostate cancer and examined its clinical implications for salvage treatment selection. Although previous studies have reported detection rates using [^18^F]PSMA‐1007 PET [9, 10, 11, 12, 13], our investigation extends these observations by characterizing recurrence site distribution in a Japanese cohort. A key finding of this study is the identification of pathological grade and PSA‐DT as independent factors associated with metastatic recurrence on PSMA‐PET.

PSA‐DT has been well established as a strong prognostic marker in patients with BCR, with shorter PSA‐DT consistently associated with an increased risk of metastatic progression detected by conventional imaging [2, 15]. In a systematic review of PSMA‐PET imaging, Perera et al. evaluated the predictive value of PSA levels and PSA‐DT for overall detection rates. The positive scan rates were 42%, 58%, 76%, and 95% for PSA categories of 0–0.2, 0.2–1, 1–2, and > 2 ng/mL, respectively. Comparable trends were observed for PSA‐DT: the pooled PSMA positivity rate was 64% for PSA‐DT ≥ 6 months and 92% for PSA‐DT < 6 months [16]. However, several studies have reported that PSA‐DT does not consistently correlate with PSMA‐PET detection rates [17, 18]. Importantly, PSA‐DT may serve a more specific role in predicting the presence of metastatic disease. Verburg et al. demonstrated that shorter PSA‐DT correlated with lymph node and bone metastases on [^68^Ga]PSMA PET [19]. These findings, together with our results, suggest that shorter PSA‐DT reflects the biological aggressiveness of recurrent disease and is associated with metastatic recurrence.

These findings are clinically significant, as both the American Urological Association and the European Association of Urology (EAU) define high‐risk BCR as a PSA‐DT of ≤ 12 months [20, 21]. In the present study, we used a cohort‐specific median PSA‐DT of 8.7 months as the primary cutoff to ensure balanced subgroup sizes and robust statistical estimation. Notably, this threshold closely corresponds to the widely applied 9‐month cutoff associated with an increased risk of prostate cancer–specific mortality [2, 22]. Furthermore, recent randomized trial evidence has demonstrated that patients with high‐risk BCR, particularly those with PSA‐DT ≤ 9 months, derive significant benefit from enzalutamide [23]. Sensitivity analyses using alternative PSA‐DT cutoffs ranging from 6 to 12 months confirmed the robustness of the association between shorter PSA‐DT and metastatic disease on PSMA‐PET (Table S3). Collectively, these findings suggest that PSA‐DT reflects the metastatic potential of recurrent prostate cancer and may serve as a pragmatic surrogate marker for systemic disease, particularly in clinical settings where PSMA‐PET is not readily accessible.

Pathological grade is also an important determinant of metastatic progression in patients with BCR. In particular, a Gleason score ≥ 8 has been correlated with metastatic progression and prostate cancer–specific mortality [2, 15]. In a systematic review of PSMA‐PET imaging, Perera et al. found no significant differences in scan positivity between Gleason scores ≤ 7 and ≥ 8 [5]. In our study, Grade Group ≥ 4 was associated with the detection of metastatic lesions, reflecting the aggressiveness of high‐grade tumors. These results align with the EAU prostate cancer guideline recommendations, which define high‐risk BCR as PSA‐DT < 12 months and Grade Group ≥ 4 [21]. Similarly, Ferdinandus et al. recently reported that patients classified as high‐risk for BCR exhibited a higher detection rate of metastatic disease on PSMA‐PET compared with those in the low‐risk group [24].

Improved detection of both local and metastatic lesions in patients with BCR of prostate cancer can substantially influence subsequent treatment selection [25, 26, 27]. The advent of PSMA‐PET imaging, particularly [^68^Ga]PSMA PET, has increased the use of lesion‐directed therapies such as radiotherapy and lymph node dissection, while reducing reliance on ADT [28]. In the phase 3 CONDOR trial, [^18^F]DCFPyL PET/CT demonstrated high lesion localization rates (85%–87%) at a median PSA level of 0.8 ng/mL and led to management changes in 64% of patients [29]. Metastasis‐directed therapy for oligometastatic disease has been shown to delay disease progression and defer systemic treatment initiation. In the phase 2 ORIOLE trial, SBRT significantly reduced 6‐month disease progression compared with observation (19% vs. 61%). Notably, patients staged with PSMA‐PET developed fewer new metastatic lesions than those staged without PSMA‐PET (16% vs. 63%) [30]. In our study, lesion‐directed therapy was selected in 46% of patients following PSMA‐PET and achieved ≥ 50% and ≥ 90% PSA reductions in 82% and 60% of cases, respectively. These findings support the role of PSMA‐PET in guiding lesion‐directed treatment strategies.

Several limitations should be acknowledged. First, this was a retrospective analysis, and [^18^F]PSMA‐1007 PET was performed as part of a clinical research protocol rather than in routine clinical practice, which may limit generalizability. Second, because histopathological confirmation or systematic imaging follow‐up were unavailable for all patients, the diagnostic accuracy of [^18^F]PSMA‐1007 PET could not be formally assessed. Accordingly, the findings should be interpreted as detection rates rather than as validated measures of sensitivity and specificity. Third, the number of patients who underwent lesion‐directed therapies following PSMA‐PET was relatively small, and treatment modalities were heterogeneous, precluding definitive conclusions regarding comparative effectiveness.

Despite these limitations, our study provides clinically significant insights into recurrence site distribution detected by [^18^F]PSMA‐1007 PET and its association with clinicopathological risk factors and salvage treatment outcomes in patients with BCR. These findings indicate that PSMA‐PET may play a pivotal role in risk stratification and individualized salvage treatment planning by differentiating localized from metastatic recurrence. Prospective studies with standardized treatment protocols are warranted to validate these findings and to elucidate the long‐term clinical impact of PSMA‐PET–guided management.

## Author Contributions

**Masatoshi Konishi:** data curation. **Tomohiro Kanaki:** data curation. **Taigo Kato:** data curation. **Yu Ishizuya:** data curation. **Atsunari Kawashima:** data curation. **Takuji Hayashi:** data curation. **Koji Hatano:** conceptualization, data curation, formal analysis, funding acquisition, investigation, methodology, project administration, visualization, writing – original draft, writing – review and editing. **Tadashi Watabe:** conceptualization, data curation, formal analysis, methodology, project administration, writing – review and editing. **Masaru Tani:** data curation. **Frederik L. Giesel:** project administration, supervision. **Takero Hirata:** data curation, methodology. **Norio Nonomura:** project administration, supervision, writing – review and editing. **Kazutoshi Fujita:** data curation. **Yoshiyuki Yamamoto:** data curation. **Motohide Uemura:** data curation. **Toshiki Oka:** data curation.

## Funding

This work was supported by the Japan Society for the Promotion of Science KAKENHI (25 K02772).

## Ethics Statement

This study was approved by the Institutional Review Board of Osaka University Hospital (Approval Nos. 19 066 and 13 397–24).

## Consent

All participants received detailed information about the study and provided written informed consent prior to participation.

## Conflicts of Interest

Kazutoshi Fujita and Norio Nonomura serve as editorial board members of the International Journal of Urology and are co‐authors of this article. To minimize potential bias, they were not involved in any editorial decisions concerning the review or acceptance of this manuscript. Frederik Giesel is an advisor at ABX, SOFIE Biosciences, Telix Pharma, Rhine pharma, and α‐Fusion and has a patent application for PSMA‐1007. All other authors declare no conflicts of interest.

## Supporting information


**Table S1:** Number of patients with lesions classified by PSA value, source data for Figure 2c.
**Table S2:** Univariate logistic regression analysis for predicting metastasis using continuous variables.
**Table S3:** Association between PSA‐DT and metastatic disease.

## Data Availability

The data that support the findings of this study are available on request from the corresponding author. The data are not publicly available due to privacy or ethical restrictions.
