# Supplementary material for: [ 18F]PSMA‐1007 PET–Based Recurrence Site Distribution and Predictors of Metastatic Disease in Patients With Biochemical Recurrence of Prostate Cancer
Source: Int J Urol. 2026 Jul 31;33(8):e70587. doi: 10.1111/iju.70587 (PMC13425049; doi:10.1111/iju.70587)
Supplement: Supplementary file 1 — Table S1: Number of patients with lesions classified by PSA value, source data for Figure 2c. Table S2: Univariate logistic regression analysis for predicting metastasis using continuous variables. Table S3: Association between PSA‐DT and metastatic disease. [file IJU-33-0-s001.docx]

Supplementary Table 1. Number of patients with lesions classified by PSA value, source data for Figure 2 (c)

|  | Bone | Lymph node | Local | No lesion | Overall |
| --- | --- | --- | --- | --- | --- |
| PSA < 0.5 ng/mL | 3 | 4 | 6 | 14 | 27 |
| PSA 0.5-1.0 ng/mL | 8 | 7 | 5 | 2 | 22 |
| PSA > 1.0 ng/mL | 22 | 26 | 26 | 6 | 80 |

| Supplementary Table 2. Univariate logistic regression analysis for predicting metastasis using continuous variables | | | | |
| --- | --- | --- | --- | --- |
|  | OR | 95% CI | | P value |
|  |  | Lower | Upper |  |
| Age | 0.98 | 0.93 | 1.03 | 0.38 |
| PSA value at BCR (ng/mL) | 1.04 | 0.96 | 1.15 | 0.38 |
| PSA-DT at BCR (months) | 0.85 | 0.79 | 0.90 | <0.001 |

BCR, biochemical recurrence; CI, confidence interval; OR, odds ratio; PSA, prostate specific antigen; PSA-DT, PSA-doubling time.

Supplementary Table 3. Association between PSA-DT and metastatic disease.

| PSA-DT cutoffs (months) | n | Metastatic disease n (%) | P value |
| --- | --- | --- | --- |
| < 6 vs ≥ 6 | 48 vs 81 | 40 (83) vs 30 (37) | P<0.001 |
| < 7 vs ≥ 7 | 55 vs 74 | 46 (84) vs 24 (32) | P<0.001 |
| < 8 vs ≥ 8 | 60 vs 69 | 50 (83) vs 20 (29) | P<0.001 |
| < 9 vs ≥ 9 | 69 vs 60 | 54 (78) vs 16 (27) | P<0.001 |
| < 10 vs ≥ 10 | 76 vs 53 | 58 (76) vs 12 (23) | P<0.001 |
| < 11 vs ≥ 11 | 79 vs 50 | 60 (76) vs 10 (20) | P<0.001 |
| < 12 vs ≥ 12 | 80 vs 49 | 61 (76) vs 9 (18) | P<0.001 |
| PSA-DT; prostate specific antigen-doubling time | | | |
